# Therapeutic Prospects of Exon Skipping for Epidermolysis Bullosa

**DOI:** 10.3390/ijms222212222

**Published:** 2021-11-12

**Authors:** Franciscus C. Vermeer, Jeroen Bremer, Robert J. Sietsma, Aileen Sandilands, Robyn P. Hickerson, Marieke C. Bolling, Anna M.G. Pasmooij, Henny H. Lemmink, Morris A. Swertz, Nine V.A.M. Knoers, K. Joeri van der Velde, Peter C. van den Akker

**Affiliations:** 1 University of Groningen, University Medical Center Groningen, Department of Genetics, 9700 RB Groningen, The Netherlands; f.c.vermeer@umcg.nl (F.C.V.); j.bremer@umcg.nl (J.B.); work.robertsietsma@gmail.com (R.J.S.); h.h.lemmink@umcg.nl (H.H.L.); m.a.swertz@gmail.com (M.A.S.); v.v.a.m.knoers@umcg.nl (N.V.A.M.K.); joeriv@gmail.com (K.J.v.d.V.); 2 University of Groningen, University Medical Center Groningen, Department of Dermatology, 9700 RB Groningen, The Netherlands; m.c.bolling@umcg.nl (M.C.B.); am.pasmooij@cbg-meb.nl (A.M.G.P.); 3 University of Groningen, University Medical Center Groningen, Genomics Coordination Center, Antonius Deusinglaan 1, 9713 AV Groningen, The Netherlands; 4 Division of Biological Chemistry and Drug Discovery, School of Life Sciences, University of Dundee, Dundee DD1 5EH, UK; a.sandilands@dundee.ac.uk (A.S.); r.p.hickerson@dundee.ac.uk (R.P.H.)

**Keywords:** epidermolysis bullosa, exon skipping, antisense oligonucleotide

## Abstract

Epidermolysis bullosa is a group of genetic skin conditions characterized by abnormal skin (and mucosal) fragility caused by pathogenic variants in various genes. The disease severity ranges from early childhood mortality in the most severe types to occasional acral blistering in the mildest types. The subtype and severity of EB is linked to the gene involved and the specific variants in that gene, which also determine its mode of inheritance. Current treatment is mainly focused on symptomatic relief such as wound care and blister prevention, because truly curative treatment options are still at the preclinical stage. Given the current level of understanding, the broad spectrum of genes and variants underlying EB makes it impossible to develop a single treatment strategy for all patients. It is likely that many different variant-specific treatment strategies will be needed to ultimately treat all patients. Antisense-oligonucleotide (ASO)-mediated exon skipping aims to counteract pathogenic sequence variants by restoring the open reading frame through the removal of the mutant exon from the pre-messenger RNA. This should lead to the restored production of the protein absent in the affected skin and, consequently, improvement of the phenotype. Several preclinical studies have demonstrated that exon skipping can restore protein production in vitro, in skin equivalents, and in skin grafts derived from EB-patient skin cells, indicating that ASO-mediated exon skipping could be a viable strategy as a topical or systemic treatment. The potential value of exon skipping for EB is supported by a study showing reduced phenotypic severity in patients who carry variants that result in natural exon skipping. In this article, we review the substantial progress made on exon skipping for EB in the past 15 years and highlight the opportunities and current challenges of this RNA-based therapy approach. In addition, we present a prioritization strategy for the development of exon skipping based on genomic information of all EB-involved genes.

## 1. Epidermolysis Bullosa

Epidermolysis bullosa (EB) is a group of rare skin conditions caused by variants in genes coding primarily for structural proteins involved in skin integrity and cell–cell adhesion such as keratins, integrins, laminins, plakins, and collagens. Depending on the gene involved, pathogenic variants can lead to a multitude of EB subtype-specific skin and sometimes extracutaneous complications [1,2]. The four main types of EB are EB simplex (EBS), Junctional EB (JEB), Dystrophic EB (DEB), and Kindler EB (KEB), with some of the types further divided into multiple subtypes (Table 1). In EBS, blisters form in the basal keratinocyte layer of the epidermis. The most important EBS genes are *KRT5* (Keratin 5, OMIM 148040) and *KRT14* (Keratin 14, OMIM 148040), which together dimerize to form the intermediate filaments that serve as the cytoskeleton of basal keratinocytes. Structurally weakened keratinocytes are prone to damage due to mechanical friction, which in turn causes cytolysis and blistering within the basal epidermis. EBS can present in a localized, intermediate, or generalized subtype. In localized EBS (OMIM 131800), blistering is usually limited to the hands and feet. In intermediate and generalized EBS (OMIM 131760), blistering can occur all over the body, and this is accompanied by a typical inflammatory aspect and herpetiform distribution of the lesions in generalized EBS. Other rarer forms of EBS are caused by variants in the genes *KLHL24* (Kelch-like family member 24, OMIM 617294), *EXPH5* (Exophilin 5, OMIM 612878), *PLEC* (Plectin, OMIM 601282), *DST* (Dystonin, OMIM 615425), and *CD151* (Tetraspanin-24, OMIM 609057). In JEB, blistering occurs within the lamina lucida zone of the cutaneous basement membrane zone. JEB is caused by defects in laminin 332, integrin α6β4, or type XVII collagen that are caused by pathogenic variants in the *LAMA3* (Laminin α3 chain, OMIM 600805), *LAMB3* (Laminin β3 chain, OMIM 150310), *LAMC2* (Laminin γ2 chain, OMIM 150292), *ITGA6* (Integrin α6, OMIM 226730), *ITGB4* (Integrin β4, OMIM 147557), or *COL17A1* (Type XVII Collagen, OMIM 113811) genes. These proteins facilitate the adhesion of basal keratinocytes to the collagen in the upper dermis. There are two main JEB subtypes that differ in severity: intermediate and severe JEB. Intermediate JEB (OMIM 226650) is characterized by generalized skin blistering, hypergranulation, and wound-healing problems, nail loss, and frequently permanent hair loss. In severe JEB (formerly known as Herlitz type, OMIM 226700), in addition to blistering over the entire body, mucosal membranes such as the airway and digestive tract are also affected, and patients usually die within one or two years after birth. In DEB, blistering occurs just below the lamina densa of the basement membrane zone due to pathogenic variants in *COL7A1*, which encodes the type VII collagen molecule that self-aggregates to form anchoring fibrils that secure attachment of the epidermis to the underlying dermis. DEB can be divided into dominantly (DDEB, OMIM 131750) or recessively (RDEB, OMIM 226600) inherited DEB. DEB phenotypes form a spectrum that ranges in severity from mild to severe, where RDEB is usually more severe than DDEB. The severe phenotypes of the disease are characterized by recurrent scarring of the skin and in the mouth and esophagus, and often extracutaneous complications. Furthermore, excessive scarring can lead to the fusion of fingers and toes. Eventually, patients with the more severe forms of RDEB are at a high risk of developing squamous cell carcinoma, of which they usually die before the age of 40 [3]. DDEB is mainly caused by glycine substitutions in type VII collagen in the central triple helix domain of *COL7A1* [4], which leads to impaired formation of the triple helix structure and dominant negative interference of the mutant over the wild-type allele. For RDEB patients with the most severe phenotype (RDEB-sev), the pathogenic variants underlying the phenotype are, generally, null variants, leading to a premature termination codon (PTC) and the complete absence of type VII collagen expression. The fourth and least prevalent type of EB is KEB. KEB is characterized by trauma-induced blistering, which mostly affects the extremities, skin atrophy, and poikiloderma. In addition, there is mucosal involvement and photosensitivity. It is caused by pathogenic variants in the *FERMT1* gene (OMIM 173650), encoding the kindlin-1 protein that is localized in the focal adhesions of the basal keratinocytes and plays important roles in cell adhesion and integrin signaling [5].

Curative and disease-modifying treatments for these diseases are being studied intensively [6], but no truly curative treatments have been presented yet. Due to the broad spectrum of genes and variants underlying EB, it is not possible to target every patient with a single treatment strategy. As such, effective treatments for EB will have to target patients/variants individually and will be as such highly personalized. An ideal therapy for EB would correct the patient-specific variants through, for example, genome editing strategies such as CRISPR-Cas9 [7,8,9]. Research into genome editing as a therapy for EB is currently ongoing, and researchers have reported success with genome editing of *COL7A1* [8] and *LAMB3* [10]. However, while genome editing is proven successful in vitro and ex vivo for EB, there are still hurdles to overcome [11]. Therefore, it is important to consider alternatives that can be realized on shorter timescales with wider applicability. One such approach could be exon skipping.

## 2. Exon Skipping

Antisense oligonucleotide (ASO)-mediated exon skipping aims to remove an exon that harbors a pathogenic variant from pre-messenger RNA (pre-mRNA) in order to mitigate the effects of the variant. Exon skipping is facilitated through ASOs that are designed to bind to the exon that contains the pathogenic variant and thereby mask it from the cellular-splicing machinery. The end goal of exon skipping is the production of an alternatively spliced mRNA in which the target (i.e., mutation-containing) exon is removed and the open reading frame is restored. Depending on the size of the skipped exon, the resulting protein will be shorter than wild type, which could have a negative impact on protein function compared to the wild-type protein. However, according to studies on naturally occurring exon skipping, in EB, this is still preferable to no protein being produced at all [12]. Extensive research into splice-modulating antisense oligonucleotide therapies has led to the approval of several ASO drugs by the FDA [13] and/or EMA, such as Nusinersen for spinal muscular atrophy (OMIM 253300) and Eteplirsen [14] and Viltolarsen [15] for Duchenne muscular dystrophy (OMIM 310200). Preclinical research into exon skipping for *COL7A1* and *COL17A1* has demonstrated its potential as therapy for EB as well [16,17,18,19].

## 3. Antisense Oligonucleotides

ASOs are small (≈15−25 nucleotides) single-stranded molecules of RNA designed to hybridize to specific messenger RNAs. Two classes of antisense mechanisms can be distinguished depending on their mode of action. The first class of ASOs (RNA–DNA hybrid ASOs) bind and degrade the target mRNA through RNase-H mediated degradation pathways. The second class of ASOs are splice switching ASOs, which affect pre-mRNA splicing through the steric hindrance of small nuclear ribonucleoproteins [20]. Compounds such as Inotersen act through the first mechanism category by targeting the *TTR* gene involved in hereditary transthyretin-mediated amyloidosis (OMIM 105210), leading to the degradation of mutant mRNA. In contrast, compounds such as Nusinersen, which acts upon the *SMN-2* gene involved in spinal muscular atrophy, and Golodirsen, which targets *DMD* for Duchenne muscular dystrophy, are examples of the second mechanism class of ASOs and act through steric blocking in order to induce alternative splicing [21]. ASO-mediated exon skipping for EB falls into this second category.

**Figure 1:**
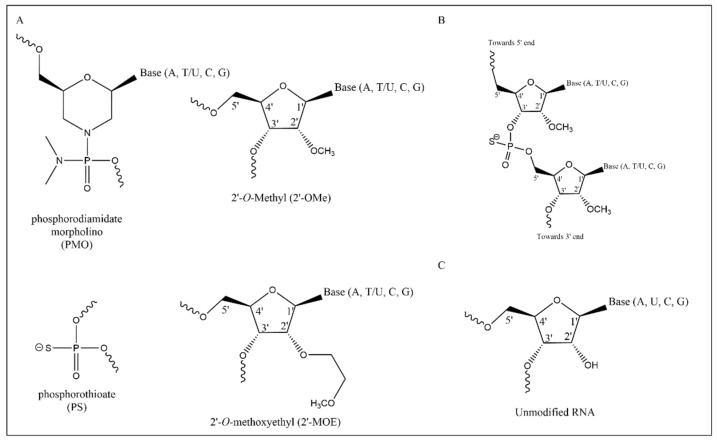
ASO chemistries (**A**) Different chemical modifications of the nucleotide ribose-ring (‘chemistry’) used in EB exon skipping ASOs. Curved lines indicate the place of linkage to other nucleotides. (**B**) Schematic representation of two 2′-OMe-PS modified RNA-nucleotides. (**C**) An unmodified RNA structure.

ASO design plays a crucial role in the overall efficacy of exon skipping therapy. Important factors that must be considered and optimized before exon skipping therapies can be considered for trials in patients include the following: the nature of the chemical backbone and nucleotide modifications, the specific ASO sequence, and the choice of delivery method. Many different chemical modifications of the nucleotide′s ribose have been developed. To date, studies on exon skipping for EB have all used ASOs containing 2′-*O*-methyl modified bases (2′*O*Me) on a phosphorothioate (PS) backbone (2′*O*Me-PS), 2′-*O*-methoxyethyl (2′-MOE)-modified bases with phosphorothioate backbone (2′-MOE-PS), and phosphorodiamidate morpholino oligomer (PMO) [16,17,19,22] (Figure 1). These ASO chemistries have been tested in vivo [21,23], and each can confer different benefits to pharmacokinetics and pharmacodynamics [21]. For example, the PS-backbone modification confers nuclease resistance, reduced hydrophilicity, and enhanced protein binding, but it is also known to carry a risk of coagulation inhibition and activation of the complement immune system [24]. 2′-*O*-methoxyethyl (2′-MOE) has a lower chemistry-specific toxicity compared to 2′-*O*-methyl (2′-*O*Me), while PMO has a somewhat lower potency compared to other chemistries but a generally low safety risk and high stability [23,25,26]. Therefore, careful consideration and analysis of the ASO chemistry for specific targets is essential. However, it is difficult to directly compare the performance of different chemistries, as delivery requirements can be chemistry-dependent [22].

## 4. ASOs Targeting Mutated Exons in Epidermolysis Bullosa

Proof-of-concept studies have demonstrated the feasibility of the exon skipping approach for *COL7A1* and *COL17A1* (Table 2). Since 91% of its exons are in-frame and potentially skippable, the early focus of exon skipping in EB has been on *COL7A1*, such as by Goto et al., where the skipping of exon 70 of *COL7A1* was induced using a 2′-*O*Me-PS ASO [27]. Protein restoration, although at low efficiency (6% using IF), was observed through immunofluorescence (IF) in primary RDEB keratinocytes transfected with ASOs and in human skin equivalents (HSE), mimicking the characteristics of human skin. HSEs were grown from RDEB patient keratinocytes and fibroblasts and were grafted on rats treated with a single local injection of ASOs (30 μg) subcutaneously below graft. Exon 73 of *COL7A1* is a hotspot for variants that underlie RDEB and DDEB [28] and is therefore an interesting target for exon skipping therapy. QR-313 (Wings Therapeutics) is a 21 bp 2′-MOE-PS ASO designed for the skipping of *COL7A1* exon 73 [17] that was in a phase 1/2 clinical trial for RDEB and DDEB (trial was terminated due to low patient enrollment). QR-313 was shown to efficiently skip exon 73 in vitro and facilitate the synthesis of type VII collagen in exon 73 mutated fibroblasts and keratinocytes (≈30−35% efficiency in ddPCR analysis). QR-313, formulated in a carbomer hydrogel, was also applied topically to wound beds in concentrations up to 200 nM in tape-stripped HSEs generated from both wild-type and mutated exon 73 fibroblasts and keratinocytes. Using digital droplet PCR (ddPCR), exon 73 skipping efficiency after 2 weeks of treatment of HSEs with 50 mg/cm^2^ QR-313 formulation was increased by ≈30−35% compared to control in the wounded dermis of the RDEB HSEs. Other studies have demonstrated exon skipping for *COL7A1* exons 10, 73, 80, and 105 [16,18,22,27,29].

In a separate study that targeted exon 73 and exon 80, 2′-*O*Me ASOs designed for exon 80 reached up to 90% in primary cultured RDEB fibroblasts and 50% skip efficiency in keratinocytes when assaying skip efficiency using RT-PCR and sequencing 48 h after transfection [18]. For exon 73, efficiency was observed to be around 95% for primary fibroblasts and 60% for keratinocytes. ASOs were supplied to these cells in doses of 50 nM, 100 nM, and 200 nM. Protein restoration levels in patient RDEB keratinocytes at the 50 nM dosage reached 32% of type VII collagen expression in Western blot analysis, whereas this was 19% for exon 80 [18]. Type VII collagen expression restoration was also observed by IF nude mice xenografts when subcutaneously injected with 1 × 500 μg or 2 × 1000 μg exon 73 skipping ASO, or 1 × 200 μg or 2 × 400 μg exon 80 skipping ASO directly under the graft.

In a separate study regarding the splicing sequence of *COL7A1*, Ham et al. found that PMO and 2′*O*Me-PS ASOs developed for exon 73 and exon 10 also showed exon skipping in vitro as confirmed by Sanger sequencing for both 2′*O*Me-PS and PMO chemistries [22].

2′*O*Me-PS ASOs developed for exon 105 have also been shown to induce exon skipping in mouse xenografts [16]. Primary cultured patient fibroblasts and keratinocytes were grafted onto nude mice using a silicone grafting chamber [30]. The mice were subsequently treated with daily systemic injections of 50 mg/kg ASO 5 days a week for 8 weeks. Exon skipping was confirmed through RT-PCR and type VII collagen expression through IF staining of type VII collagen (efficiency could not be quantified).

*COL17A1* exclusively consists of in-frame exons and therefore is also attractive for exon skipping. Ablinger et al. designed ASOs against *COL17A1* exon 7 based on a patient with intermediate JEB carrying a variant in this exon. ASO treatment restored type XVII collagen expression in patient-derived primary cultured keratinocytes through exon 7 skipping [19]. The authors were also able to identify type XVII collagen expression at the cutaneous basal membrane in HSEs generated from JEB keratinocytes pretreated with *COL17A1* exon 7 ASOs. Type XVII collagen deposition was also observed in the cells treated with the ASOs.

It is difficult to compare exon skipping efficiencies reported by different studies due to the different methods and definitions used to ascertain these efficiencies. Standardization of the methods used to measure exon skipping efficiency could improve our ability to compare exon skipping efficiencies between studies and broaden our insights on what makes for an optimal ASO sequence. For the optimization of ASOs for exon skipping in Duchenne muscular dystrophy, ddPCR was recommended as the method of choice for determining exon skipping efficiency, after comparing to other quantification methods such as RT-qPCR and RNA sequencing [31,32]. ddPCR measures exon skipping efficiency as a percentage of transcripts containing skipped exons. Preclinical research using cell models is indispensable and provides a valuable indication of the success of exon skipping treatment, specifically with regard to sequence design. However, it is difficult to reflect the in vivo situation using cell models alone. Trialing ASO design, skipping efficiency, expression levels, and delivery methods in more complex models such as skin xenografts or ex vivo human skin models has the potential to better replicate the conditions of patient skin and more precisely assess treatment efficacy.

As previously discussed, the applicability of exon skipping to EB is highly dependent on the individual gene and specific details of pathogenic variants. However, the number of EB patients (DEB or other) who can benefit from exon skipping treatment is not yet known. For now, research is mostly focused on RDEB, as this is the more severe variant of DEB. In DDEB, since type VII collagen is already expressed, it is conceivable that the introduction of an exon-skipped allele in addition to wild-type and mutant alleles might even worsen the phenotype instead of mitigating it. Therefore, it is questionable whether exon skipping as a therapy would benefit DDEB patients [33]. However, in contrast, QR-313 was shown to improve epidermal attachment in DDEB skin models for exon 73 [17]. In addition, exon 73-skipped type VII collagen only rarely interacted with wild-type type VII collagen, which means it did not negatively impact wild-type type VII collagen function but rather added to it. It remains to be seen whether this effect is specific to the large exon 73 or if it holds true for all in-frame exons in *COL7A1*. It also remains unknown whether the phenotypic improvement is the same in vivo. If exon skipping proves to be a viable treatment strategy for DDEB, the number of patients who could benefit from exon skipping therapy would significantly increase.

## 5. Lessons from Natural Exon Skipping in Epidermolysis Bullosa

Natural exon skipping is a phenomenon in which exons are skipped due to the pathogenic variants in a gene. Several case reports have shown that natural exon skipping variants can lead to a milder type of EB than would be expected from the genotype. As the goal of ASO-mediated exon skipping therapy is to improve the phenotype of EB to a milder one, the potential therapeutic effect of ASO-mediated exon skipping can be appreciated by looking at the phenotypes of patients carrying these natural exon skipping variants. Taking *COL7A1* as an example, the difference in the way each variant leads to the expression of a phenotype can provide insight into if and how an exon skipping therapy can improve a phenotype. With this goal in mind, a recent study specifically investigated natural exon skipping variants in a cohort of 100 DDEB and 76 RDEB patients [33]. In these patients, 26 were found to carry variants in putative splice sites, and seven of these variants were found to induce in-frame exon skipping. Another 20 *COL7A1* natural exon skipping variants were found in the literature. DDEB natural exon skipping variants caused phenotypes resembling well-known DDEB phenotypes. In contrast, RDEB patients carrying natural exon skipping variants were found to have generally milder phenotypes on the RDEB spectrum than expected from their genotypes, with the exact phenotype dependent on the level of type VII collagen expression. Therefore, it is predicted that the expression of a shortened type VII collagen due to ASO-mediated exon skipping is more beneficial to the patient than a complete absence of type VII collagen, showing that ASO-mediated exon skipping is a viable treatment option for RDEB [12]. As discussed above, whether this is also true for DDEB remains unclear.

A patient with a paternal *COL7A1* exon 76 PTC variant (c.6311_6312delCT) and a maternal *COL7A1* exon 15 PTC (c.2005C>T) variant, leading to RDEB-sev in other patients, was shown to have a milder-than-expected phenotype. An additional variant (c.1907G>T) on the maternal allele, in cis with the c.2005C>T variant, weakened the exon 15 acceptor splice site and induced in-frame skipping of exon 15 that resulted in the expression of a shortened type VII collagen. Immunofluorescent staining showed lower expression levels of type VII collagen (11%) compared to control, but even these levels were sufficient to attenuate the phenotype toward a milder form. Other examples of natural exon skipping are patients with RDEB and JEB carrying predicted PTC variants in *COL7A1* and *LAMB3*, respectively [34]. However, these variants did not behave as null variants and instead induced natural exon skipping and hence milder-than-expected phenotypes. The *COL7A1* variant c.2471dupG p.(Asn825Lysfs*41) and the *LAMB3* variant c.2500C>T p.(Gln834*) both occurred in purine-rich regions that may act as splicing enhancers. The proposed mechanism was that the disruption of exonic splicing enhancer activity by these variants led to natural exon skipping, which bypassed the PTCs and restored the reading frame i.e., exactly the mechanism by which ASO-mediated exon skipping aims to act.

Another example of natural exon skipping was observed in a patient with JEB intermediate who was compound heterozygous for PTC variants in exon 18 and 49 of *COL17A1* [35]. Several non-blistering, healthy skin patches were observed on the patient′s arm. In IF staining with three antibodies for type XVII collagen, a mosaic pattern was observed. Small patches of the basal membrane zone resembled human normal control skin, as visualized using exon 7 and exon 47–48 antibodies, while staining with an exon 48–49 antibody was completely negative. Selectively isolated type XVII collagen-positive keratinocytes showed a reverse mutation in the exon 49 acceptor splice site that induced the skipping of this exon and restoration of type XVII protein production.

Another case report studied a patient with EBS with pyloric atresia (EBS-PA) caused by a maternal *PLEC* null variant (c.7396C>T) in exon 31 and a paternal null variant (c.7633C>T) in exon 32 [36]. Whereas typical EBS-PA cases show a severe and lethal phenotype, this case showed a milder phenotype with clinical improvement. While the specific mechanism behind this phenomenon was not fully understood, it was presumed that the basis of this milder phenotype lies in alternative splice variants of the *PLEC* transcript. RNA analysis of *PLEC* in various human control tissues showed the presence of alternatively spliced isoforms that were missing the rod domain encoded by exon 31 [36]. Deletion of the plectin rod domain in C57Bl/6 mice revealed that the rod domain was not essential for the tissue integrity function of plectin [37].

Together, these reports make the case for exon skipping as a promising treatment strategy for EB patients with homozygous null variants. However, it remains elusive whether exon skipping therapy would be suitable for treating dominant EB types. For example, a 30 bp deletion in intron 6 of the *KRT5* gene leading to in-frame skipping of exon 6 was identified in a patient with EBS severe [38]. Exon 6 skipping led to deletion of the highly conserved 2B domain, with the result that the keratin 5 protein acted in a dominant negative fashion. Therefore, it seems unlikely that the introduction of an exon 6 skipped keratin 5 protein using ASOs would be beneficial for patients carrying dominant *KRT5* variants.

## 6. Selection of Epidermolysis Bullosa Genes and Exons for Targeting with Exon Skipping

**Figure 2:**
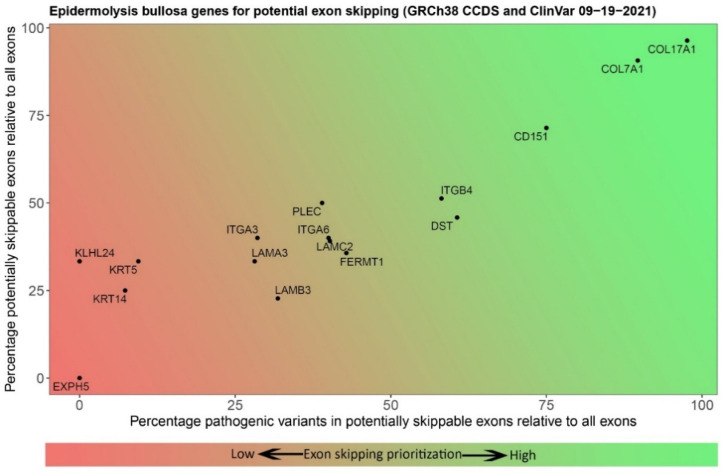
Prioritization tool for exon skipping development for EB. Visualization of the relative number of in-frame exons per EB-gene (Y-axis) and pathogenic variants (ClinVar) contained in them (X-axis). The further an exon lies within the green area, the greater the percentage of in-frame exons in that gene and the higher the percentage of variants located in those in-frame exons. This indicates that the gene is considered a good candidate for exon skipping development.

**Figure 3:**
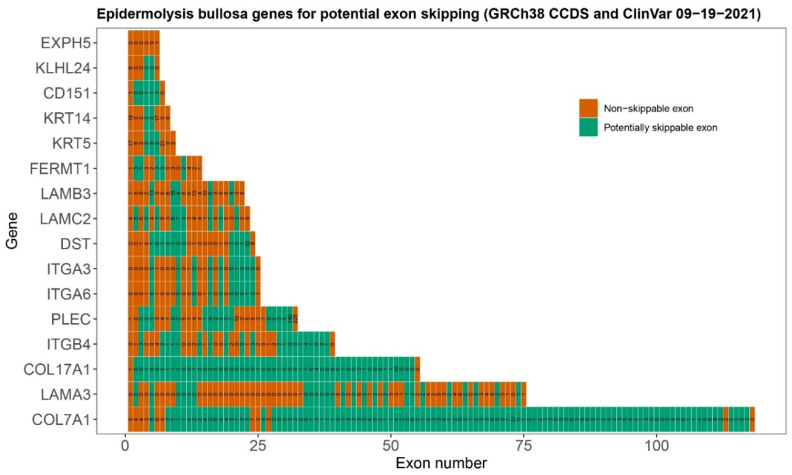
Overview of pathogenic variants per exon for each EB gene. For each EB gene (Y-axis), each exon is shown as a separate tile (not to scale, X-axis). The number of tiles indicates the absolute number of exons per gene. Green tiles represent in-frame and potentially skippable exons, red tiles represent non-skippable (out-of-frame) exons. The number in each tile is the number of pathogenic variants as recorded in ClinVar on 7 October 2021. For this figure, the canonical consensus coding sequences are used as found in Ensembl.

Selecting a gene and exon for the development of an exon skipping therapy comes with many unanswered questions. To start, the exact numbers of patients and specific variants may not always be known because we still lack a global EB database. Therefore, it is difficult to estimate how many patients would benefit from the development of exon skipping therapy for any given exon. There are also currently 16 known EB genes [2] (Table 1). The large number of genes, and the number of exons within them, requires a target prioritization method for selecting the best gene and exon. In-frame exons that have not been found to contain pathogenic variants can be excluded. Consequently, all in-frame exons that do contain pathogenic variants can be considered potential exon skipping targets. In addition, as discussed above, it is expected that exon skipping therapy would be better suited to treat bi-allelic null variants of EB genes within in-frame exons [33]. Therefore, comparison of the relative in-frame exon count of a gene, and the number and frequency of pathogenic variants in those in-frame exons may serve as a first indicator of the potential of an exon skipping strategy, because the number of times a variant has been reported can relate back to the number of patients. Thus, developing ASOs for these exons may benefit a greater number of patients. Figure 2 and Figure 3 provide a basic prioritization strategy based on the genomic and mutational characteristics of the EB genes. These preliminary characteristics provide only the first step in gene and exon selection. They do not take ASO design or protein function into account; furthermore, complexity is introduced through different isoforms of proteins. PLEC possesses eight isoforms where the first exon is different in each transcript, and the exon count of all non-coding exons comes down to 39 exons.

**Figure 4:**
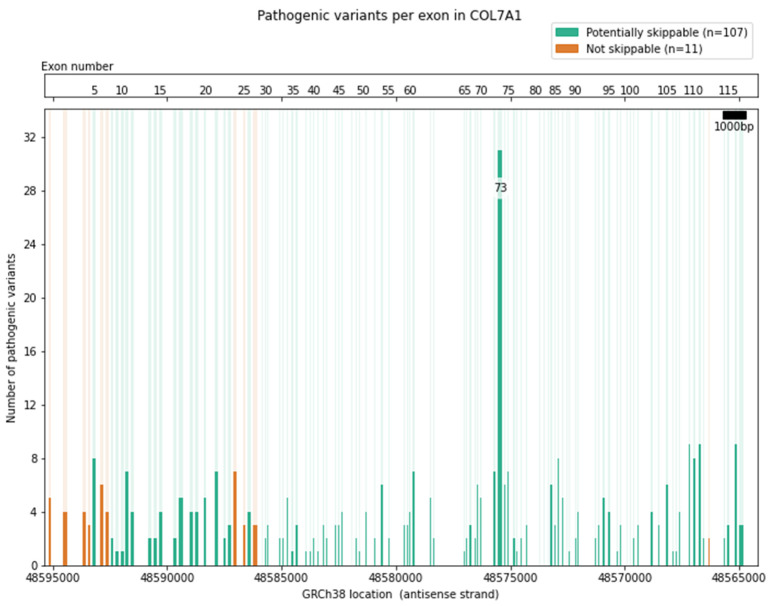
Schematic representation of the genomic structure of COL7A1 and pathogenic variants per exon. Light-green bars indicate in-frame (potentially skippable) exons, light-orange bars represent out-of-frame (non-skippable) exons. Exons are depicted to scale, the scale bar represents 1000 bps. Projected on top of each exon as dark-green and dark-orange bars are the number of pathogenic variants per exon, the height of each bar indicates the number of variants per exon. The top bar contains the exon numbers.

Some in-frame exons may be amenable to exon skipping, but these may also encode essential protein domains that are indispensable for proper protein function, effectively making them un-skippable. Therefore, exons of interest must always be considered with keeping such limitations in mind. For example, at only 31.2 kb in length with 118 exons, the *COL7A1* gene is relatively compact (Figure 4): 107 (91%) of these exons are in-frame and potentially skippable, and 270 pathogenic variants are located in these in-frame exons, as recorded in ClinVar (https://www.ncbi.nlm.nih.gov/clinvar/) accessed on 7 October 2021. However, the process does not stop there. A large part of *COL7A1* also consists of repeating elements, which could complicate the design of exon-specific ASOs. On the other hand, it could be argued that the high repetitiveness of these exons means that the absence of one exon would not have a large impact on the function of the protein as a whole [29]. A further complication in ASO design for *COL7A1* are the guanine nucleotide repeats in the collagen triple-helix domain, with a high GC percentage limiting the locations for which ASOs can be specifically designed (see supplement for other EB genes).

## 7. Development of ASOs for Targeting Exon Skipping in Epidermolysis Bullosa

The development of an exon skipping ASOs starts with identifying the target sequence. Several important criteria that need to be considered when designing an ASO sequence have emerged from previous studies [21,39,40]. In a study examining effective ASO sequence design, in vitro analysis of splice-modulating ASOs showed that effective ASOs are located closer to the acceptor splice site [40]. To further increase the exon skipping efficiency, ASOs should target sequences important to splice regulation, such as exon splice enhancers, and contain a relatively high (>50% [39]) GC-content. Nearest neighbor melting temperature should be considered to increase the stability. Finally, the ability of an ASO to form secondary structures should be investigated, because these could interfere with binding to the mRNA [40]. Exclusion criteria such as stretches of three or more G or C nucleotides should also be taken into account. These are generally avoided in ASO sequences because they risk binding too strongly to serum proteins and off-target sites [41]. Based on these criteria, in silico predictions can be made about the most efficient ASOs. However, whether these predicted ASOs are indeed the most efficient ASOs can only be determined through a laborious empirical process of trialing many different sequences. Thus far, this is the method by which all ASOs used in preclinical studies for EB have been designed. A newly developed in silico tool may help broaden our perspective in this process.

eSkip-finder is a recently published online machine-learning based tool for finding optimal ASO sequences for exon skipping [42]. To understand the power of eSkip-Finder in designing ASOs for EB, we compared the sequences of ASOs designed by ourselves and other groups based on in silico predictions and determined by empirical studies to the optimal ASO sequences predicted by eSkip-Finder. Comparisons between predicted and published sequences targeting *COL7A1* and *COL17A1* provided remarkably different results (Table 2): eSkip-Finder predicted that the ASOs designed in the classical way should have low exon-skipping efficiency, while other sequences that were avoided during classical design for reasons such as G/C triplicates were predicted to have much higher efficiency. However, the ASO sequences predicted by eSkip-Finder are a machine-learning prediction and only account for predicted skip efficiency. The tool does not account for whether an ASO can actually be synthesized or for the inclusion of guanine or cytosine repeats. Nonetheless, the optimal ASOs predicted by eSkip-Finder are worth exploring during the design of new ASOs for exon skipping to be trialed next to classically designed ASOs in order to test whether any further optimizations with regard to skipping efficiency for EB can be achieved.

Targeting specific exons for skipping is challenging, as reported by Ham et al. [22]. *COL7A1* splice sites are not strongly defined. ASOs for *COL7A1* designed to target exon 73 led to aberrant splicing of the mRNA, leading to the inclusion of intron 76 into the mature RNA. The mechanism behind this intron retention is not yet fully understood. However, it indicates that it is crucial to experimentally verify that ASOs produce the desired results in a laboratory setting, which is an additional criterion to consider in the design of ASO sequences.

## 8. Delivery of ASO Therapies

Exon skipping as a treatment is limited in that it would require repetitive treatments due to the transient nature of ASOs. Therefore, it is important to carefully consider the way in which ASO therapies are delivered in vivo. The topical application of ASOs to wound beds using carbomer hydrogel in vitro and in ex vivo porcine skin [17] or liposomes in vitro [19], has been shown to induce efficient exon skipping in cells at the site of application. The administration of ASO to the wound bed has the increased benefit of directly being applied to the exposed cells where exon skipping should be induced. However, the topical application of ASOs is not a systemic treatment. While improved wound healing and skin stabilization would improve the quality of life for patients, a systemic treatment has the potential to prevent blistering through skin stabilization in addition to treating other (mucosal) tissues inaccessible to topical treatment. Systemic treatment has been shown to work in xenograft mice [16], but the delivery of a systemic treatment does provide a challenge.

Liposomes are not a feasible strategy for systematic delivery given the possibility of non-specific uptake in different tissues. Furthermore, ASOs applied in a systemic fashion will also have to contend with difficulties such as the potential for systemic and local adverse events, such as the accumulation of ASOs in unintended tissues leading to proteinuria, inflammatory responses, and inhibition of coagulation or thrombocytopenia [21]. The exact chemical modifications of ASOs also play an important role in the choice of delivery, which is out of the scope of this review. Therefore, more research into delivery methods and ASO chemistry is crucial to target a systemic treatment specifically to the keratinocytes and fibroblasts. To solve the systemic delivery issue, the development of a basal keratinocyte-specific marker molecule may be required. This concept has been shown before using GalNac conjugates for targeting hepatocytes [43], and multiple ASO-GalNac conjugate-based treatments have been used in trials [44]. A notable example of an approved drug using the GalNac conjugate system includes Givosiran, which is a GalNac-siRNA conjugate targeting *ALAS1* in order to treat acute hepatic porphyria [45].

## 9. Concluding Remarks and Future Perspectives

While exon skipping as a treatment for genetic diseases is an established strategy, it is still in a maturing phase with regard to EB. Our review provides a perspective on the application of exon skipping as a treatment strategy for EB and provides insight into the criteria required for the development of these novel therapies. The groundwork for exon skipping has been laid, and current research is moving more toward clinical applications. ASO-mediated exon skipping as a treatment strategy for EB has a promising outlook, especially for RDEB and JEB. Exon skipping therapy also offers a more promising short-term perspective compared to treatment strategies such as genome editing. Favorable results in studies pertaining to ASO development for the exon skipping of *COL7A1* and *COL17A1* may translate to an increased understanding of the applications of exon skipping for EB in general, and the streamlined development of ASO-mediated exon skipping therapies for other forms of EB. However, while ASO-mediated exon skipping for EB is about to make the jump to the clinical setting, there are still some challenges to overcome that require further study such as the in vivo effect of EB exon skipping therapy. Furthermore, ASOs for exon skipping in EB need to be developed in a mutation-specific manner. The small cohort of patients who could benefit from a specific ASO treatment makes it commercially uninteresting to develop. On the other hand, patients who can be treated with ASOs can have a vastly improved quality of life. In addition, bringing exon skipping for EB to clinical practice using the classic therapy development route is impossible, as any trials will be small scale or even *n* = 1. Fortunately, alternative N-of-1 routes to bring such small-scale treatments to patients are being explored increasingly at the moment [46]. An example of an ASO developed in this way is Milasen, which was specifically developed for a single patient suffering from neuronal ceroid lipofuscinosis [47]. The implementation of regulations that include special considerations for individualized treatments will hopefully pave the way for developing such n-of-1 therapies for patients who would otherwise go untreated, such as exon skipping for EB [46].

## Figures and Tables

**Table 1:** Numbers of potentially skippable exons and pathogenic variants in those exons for each gene known to be involved in Epidermolysis Bullosa.

Level of Blistering	Gene	Protein	No. of Exons	In-Frame Exons	Percentage Skippable ^1^	No. of ClinVar Variants ^2^ in Skippable Exons	Percentage Variants in Skippable Exons
Basal epidermal	*KRT14*	Keratin 14	8	3	37.5	20 (39)	51.3
	*KRT5*	Keratin 5	9	4	44.4	22 (24)	45.8
	*PLEC*	Plectin	39	17	43.6	43 (49)	87.8
	*KLHL24*	Kelch-like family member 24	6	2	33.3	0 (6)	0.0
	*EXPH5*	Exophilin 5	6	0	0.0	0 (1)	0.0
	*CD151*	Tetraspanin-24	7	5	71.4	2 (2)	100.0
	*DST*	Dystonin	24	11	45.8	22 (26)	84.6
Intralamina Lucida	*COL17A1*	Type XVII Collagen	55	53	96.4	37 (38)	97.4
	*ITGB4*	Integrin β4	39	20	51.3	15 (28)	53.6
	*LAMA3*	Laminin α3 chain	75	25	33.3	24 (72)	33.3
	*LAMB3*	Laminin β3 chain	22	5	22.7	36 (108)	33.3
	*LAMC2*	Laminin γ2 chain	23	9	39.1	29 (73)	39.7
	*ITGA6*	Integrin α6	25	10	40.0	2 (4)	50.0
	*ITGA3*	Integrin α3	25	10	40.0	2 (7)	28.6
Sublamina Densa	*COL7A1*	Type VII Collagen	118	107	90.7	270 (297)	90.9
Mixed levels	*FERMT1*	Kindlin-1	14	9	64.3	12 (24)	50.0

^1^ An exon is considered skippable if the reading frame remains intact when the exon is ‘skipped’. ^2^ Only variants denoted as pathogenic in ClinVar are included (online database of variants in the human genome. Clinvar is available at https://www.ncbi.nlm.nih.gov/clinvar/, accessed on 7 October 2021). Between brackets the total number of variants in ClinVar for that gene.

**Table 2:** ASOs developed to induce exon skipping in EB genes.

Gene	Exon	ASO Sequence	GC% of ASO	ASO Length	Start Position ^1^	Predicted Efficiency % ^2^	ASO Chemistry ^3^	ASO Name	Reference
*COL7A1*	10	CGGGCCUCAGGCACCAAGUUC	66	21	64	26.0 (2′*O*Me)	2′-*O*Me-PS	H10A(+65+85)	[22]
*COL7A1*	10	CGGGCCUCAGGCACCAAGUUC	66	21	64	7.7 (PMO)	PMO	H10A(+65+85)	[22]
*COL7A1*	10	CUUCCCCCGCACUGACCAGUCUC	65	23		N.A. ^4^	2′-*O*Me-PS	H10D(+07-16)	[22]
*COL7A1*	10	CUUCCCCCGCACUGACCAGUCUC	65	23		N.A. ^4^	PMO	H10D(+07-16)	[22]
*COL7A1*	70	CGCACACUUCCAGGC	66	15	32	8.3 (2′*O*Me)	2′-*O*Me-PS		[27]
*COL7A1*	73	CGUUCUCCAGGAAAGCCGAUG	57	21	5	8.1 (2′*O*Me) ^5^	2′-MOE-PS	QR-313	[17]
*COL7A1*	73	TCTTGCGCCCGACTTCCCGCTGGCACCTCT	67	30	20	26.6 (2′*O*Me)	2′*O*Me		[18]
*COL7A1*	73	UUCAGCCCGCGUUCUCCAGG	65	20	15	11.6 (2′*O*Me)	2′-*O*Me-PS	H73A(+16+35)	[22]
*COL7A1*	73	UUCAGCCCGCGUUCUCCAGG	65	20	15	22.6 (PMO)	PMO	H73A(+16+35)	[22]
*COL7A1*	73	CGCCCUUCAGCCCGCGUUCU	70	20	20	21.3 (2′*O*Me)	2′-*O*Me-PS	H73A(+21+40)	[22]
*COL7A1*	73	CGCCCUUCAGCCCGCGUUCU	70	20	20	47.9 (PMO)	PMO	H73A(+21+40)	[22]
*COL7A1*	73	CGCCCUUCAGCCCGCGUUCUCCAGG	72	25	15	49.9 (2′*O*Me)	2′-*O*Me-PS	H73A(+16+40)	[22]
*COL7A1*	73	CGCCCUUCAGCCCGCGUUCUCCAGG	72	25	15	60.0 (PMO)	PMO	H73A(+16+40)	[22]
*COL7A1*	80	TCCCAGACGTCCCAGGTTCTCCGG	67	24		N.A. ^4^	2′*O*Me		[18]
*COL7A1*	105	GAUACCAGGCACUCCAUCCU	55	20	13	15.2 (2′*O*Me)	2′*O*Me	AON1	[16]
*COL7A1*	105	CAUGAAGCCAACAUCUCCUU	45	20	43	11.3 (2′*O*Me)	2′*O*Me	AON2	[16]
*COL17A1*	7	TTTGACTCCGTCCTCTGGTT	50	20	10	3.8 (2′*O*Me)	2′*-O*Me-PS	AON1	[19]
*COL17A1*	7	TCGTGTTTGACTCCGTCCTC	55	20	15	11.7 (2′*O*Me)	2′*-O*Me-PS	AON2	[19]
*COL17A1*	7	CTCCGTCCTCTGGTTGAAGA	55	20	5	42.4 (2′*O*Me)	2′-*O*Me-PS	AON3	[19]

^1^ Nucleotide position of first base of the target sequence as counted from the acceptor splice site in the target exon. ^2^ Exon efficiency of the ASOs as predicted by eSkip-finder in percentage of transcripts, predicted under conditions of 2′*O*Me or PMO ASOs. ^3^ Chemical modification of the ASO as described in the literature; see Figure 1A for structures. ^4^ Sequence could not be compared to eSkip-finder predictions as it was out of the prediction range. ^5^ Sequence is of the 2′MOE variant, and thus, a comparison to 2′*O*Me does not convey accurate information.

## Data Availability

Data used in the figures in this review is available at https://www.ncbi.nlm.nih.gov/clinvar and https://www.ensembl.org/index.html.

## Supplementary Materials

The following are available online at https://www.mdpi.com/article/10.3390/ijms222212222/s1.
