# Supplementary material for: Therapeutic Prospects of Exon Skipping for Epidermolysis Bullosa
Source: Int J Mol Sci. 2021 Nov 12;22(22):12222. doi: 10.3390/ijms222212222 (PMC8621297; doi:10.3390/ijms222212222)
Supplement: Supplementary file 1 [file ijms-22-12222-s001.zip › ijms-1441894-supplementary.pdf]

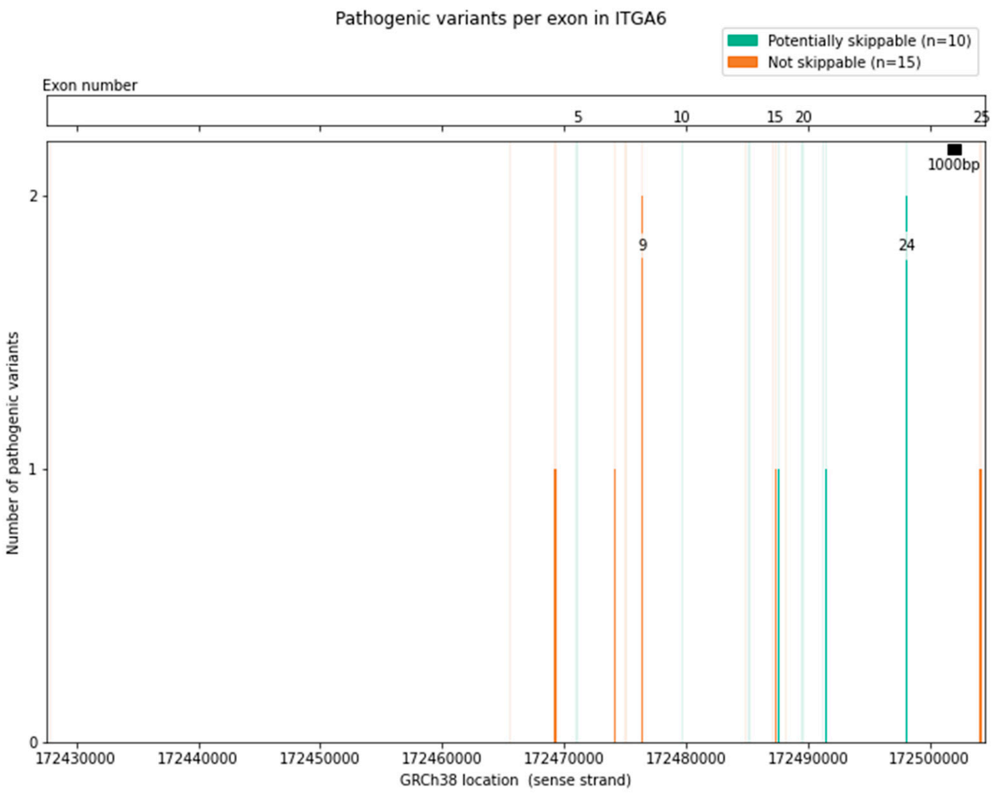

Supplemental Figure S1. Schematic representation of the genomic structure of *ITGA6* and pathogenic variants per exon. Light-green bars indicate in-frame (potentially skippable) exons, light-orange bars out-of-frame (non-skippable) exons. Exons are depicted to scale, scale-bar represents 1000 bps. Projected on top of each exon as dark-green and dark-orange bars are the number of pathogenic variants per exon, the height of each bar indicates the number of variants per exon. In the top bar the exon numbers.

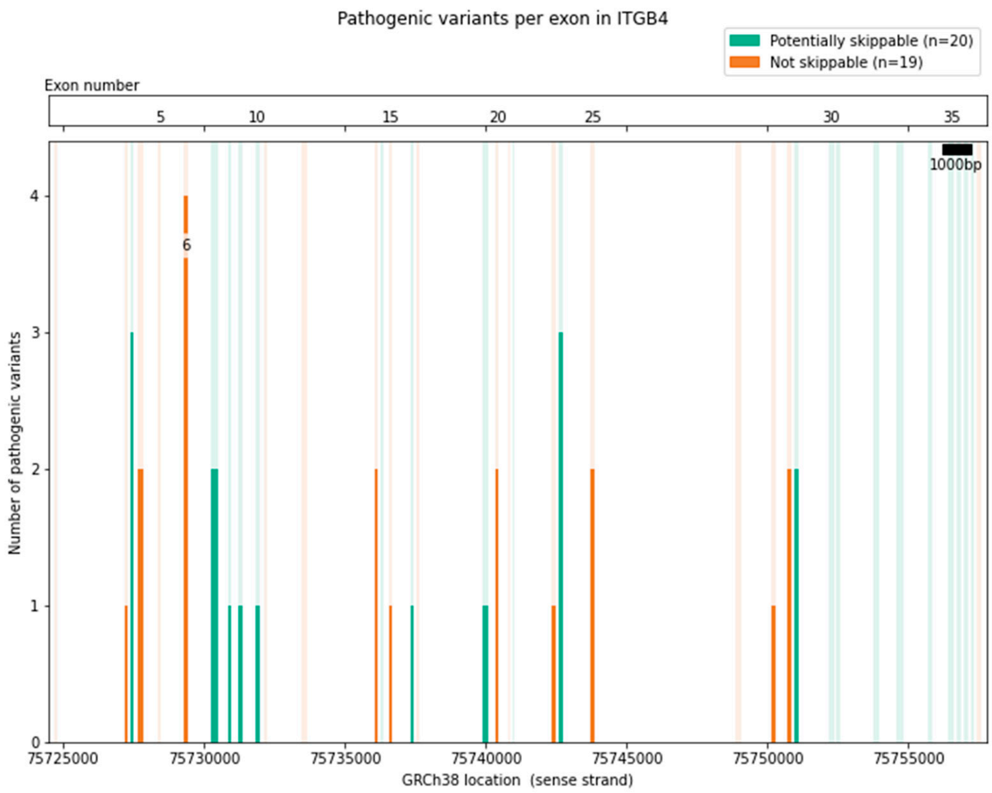

Supplemental Figure S2. Schematic representation of the genomic structure of *ITGB4* and pathogenic variants per exon. Light-green bars indicate in-frame (potentially skippable) exons, light-orange bars out-of-frame (non-skippable) exons. Exons are depicted to scale, scale-bar represents 1000 bps. Projected on top of each exon as dark-green and dark-orange bars are the number of pathogenic variants per exon, the height of each bar indicates the number of variants per exon. In the top bar the exon numbers.

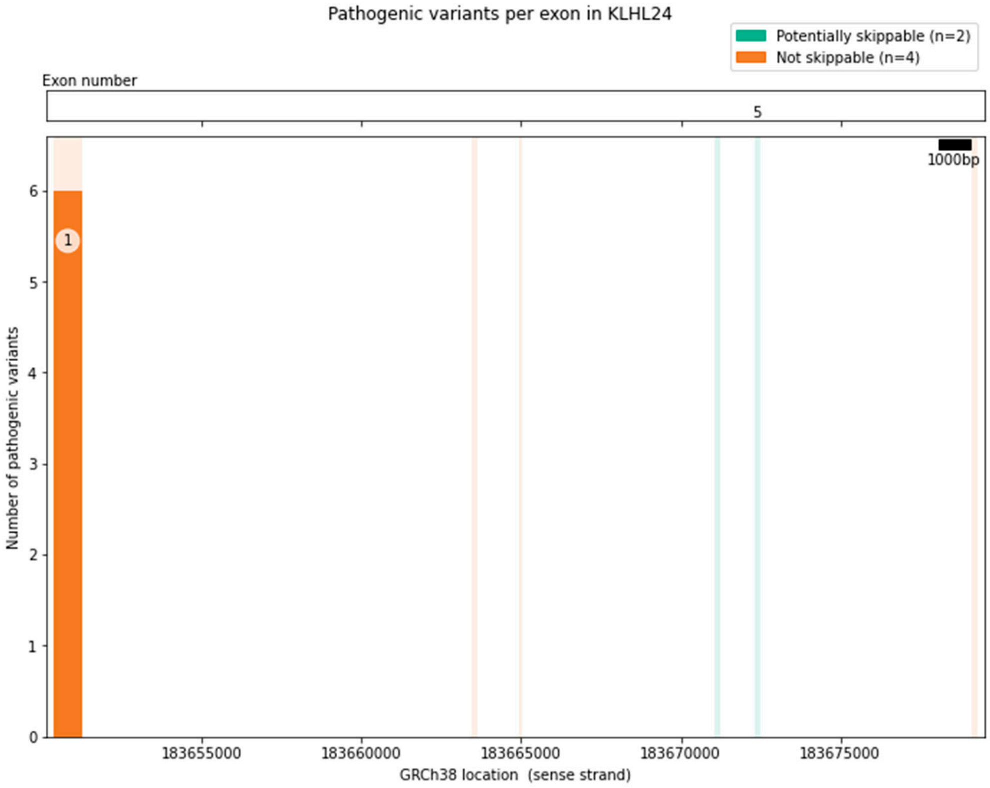

Supplemental Figure S3. Schematic representation of the genomic structure of *KLHL24* and pathogenic variants per exon. Light-green bars indicate in-frame (potentially skippable) exons, light-orange bars out-of-frame (non-skippable) exons. Exons are depicted to scale, scale-bar represents 1000 bps. Projected on top of each exon as dark-green and dark-orange bars are the number of pathogenic variants per exon, the height of each bar indicates the number of variants per exon. In the top bar the exon numbers.

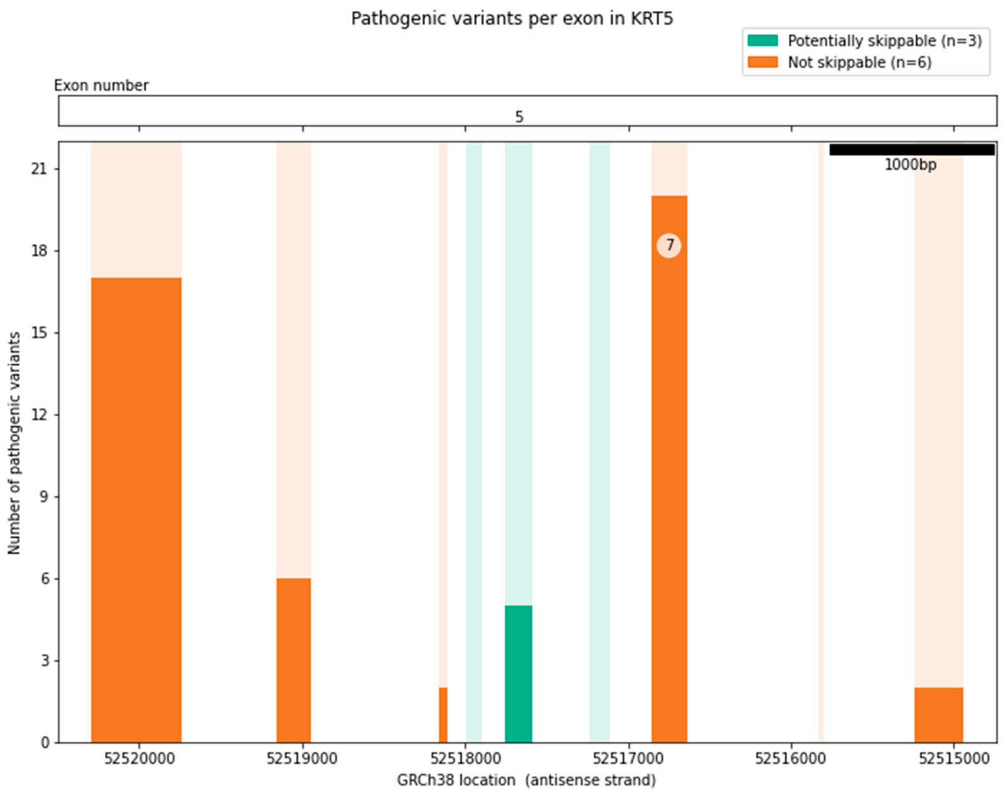

Supplemental Figure S4. Schematic representation of the genomic structure of *KRT5* and pathogenic variants per exon. Light-green bars indicate in-frame (potentially skippable) exons, light-orange bars out-of-frame (non-skippable) exons. Exons are depicted to scale, scale-bar represents 1000 bps. Projected on top of each exon as dark-green and dark-orange bars are the number of pathogenic variants per exon, the height of each bar indicates the number of variants per exon. In the top bar the exon numbers.

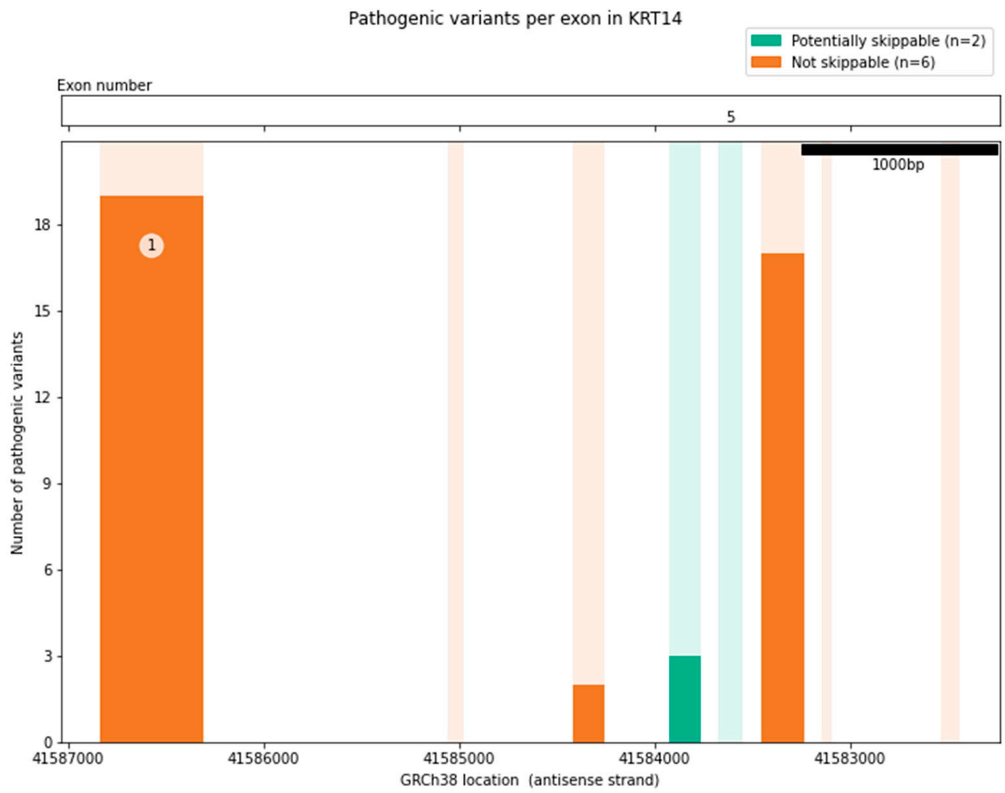

Supplemental Figure S5. Schematic representation of the genomic structure of *KRT14* and pathogenic variants per exon. Light-green bars indicate in-frame (potentially skippable) exons, light-orange bars out-of-frame (non-skippable) exons. Exons are depicted to scale, scale-bar represents 1000 bps. Projected on top of each exon as dark-green and dark-orange bars are the number of pathogenic variants per exon, the height of each bar indicates the number of variants per exon. In the top bar the exon numbers.

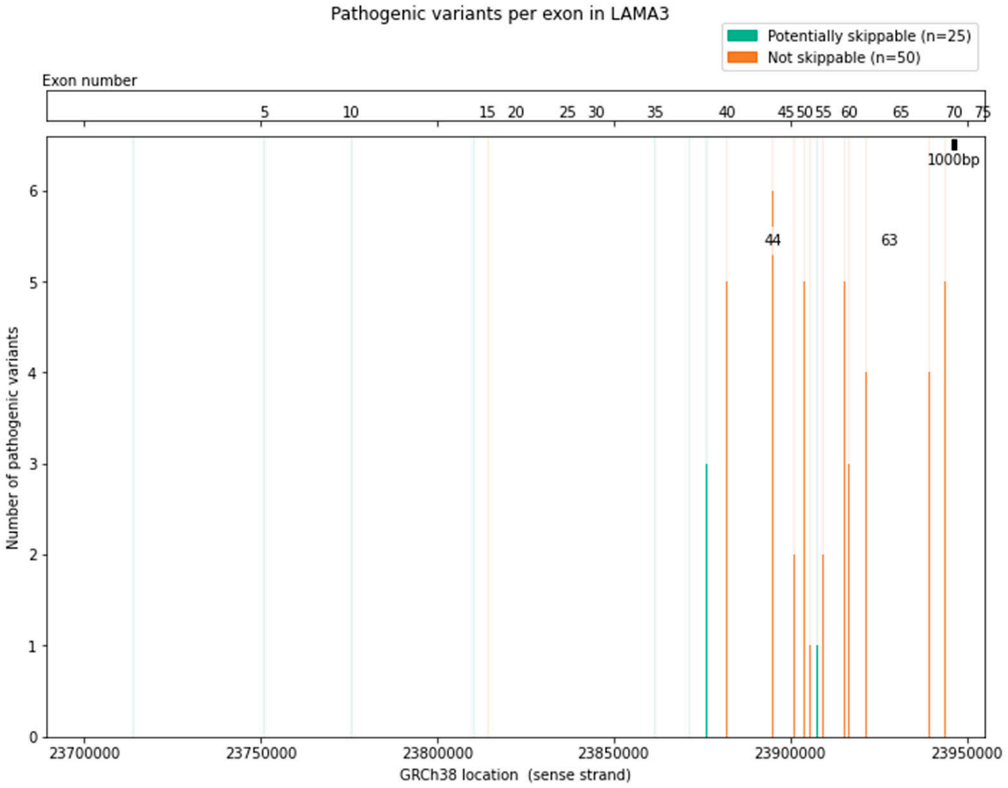

Supplemental Figure S6. Schematic representation of the genomic structure of *LAMA3* and pathogenic variants per exon. Light-green bars indicate in-frame (potentially skippable) exons, light-orange bars out-of-frame (non-skippable) exons. Exons are depicted to scale, scale-bar represents 1000 bps. Projected on top of each exon as dark-green and dark-orange bars are the number of pathogenic variants per exon, the height of each bar indicates the number of variants per exon. In the top bar the exon numbers.

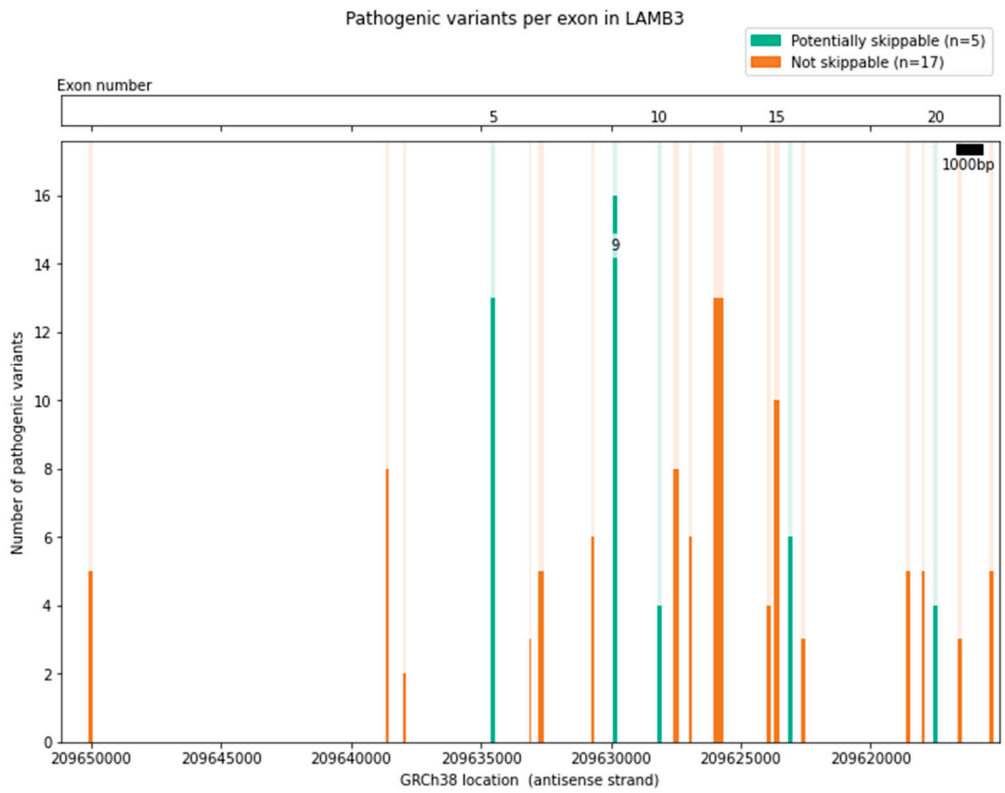

Supplemental Figure S7. Schematic representation of the genomic structure of *LAMB3* and pathogenic variants per exon. Light-green bars indicate in-frame (potentially skippable) exons, light-orange bars out-of-frame (non-skippable) exons. Exons are depicted to scale, scale-bar represents 1000 bps. Projected on top of each exon as dark-green and dark-orange bars are the number of pathogenic variants per exon, the height of each bar indicates the number of variants per exon. In the top bar the exon numbers.

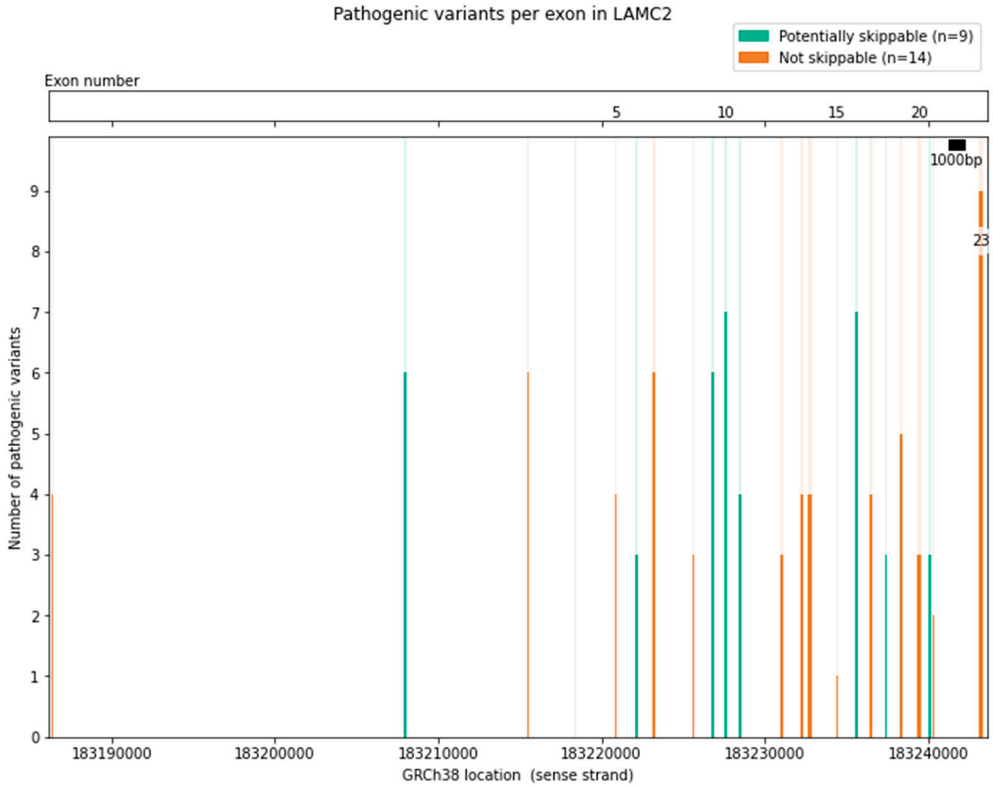

Supplemental Figure S8. Schematic representation of the genomic structure of *LAMC2* and pathogenic variants per exon. Light-green bars indicate in-frame (potentially skippable) exons, light-orange bars out-of-frame (non-skippable) exons. Exons are depicted to scale, scale-bar represents 1000 bps. Projected on top of each exon as dark-green and dark-orange bars are the number of pathogenic variants per exon, the height of each bar indicates the number of variants per exon. In the top bar the exon numbers.

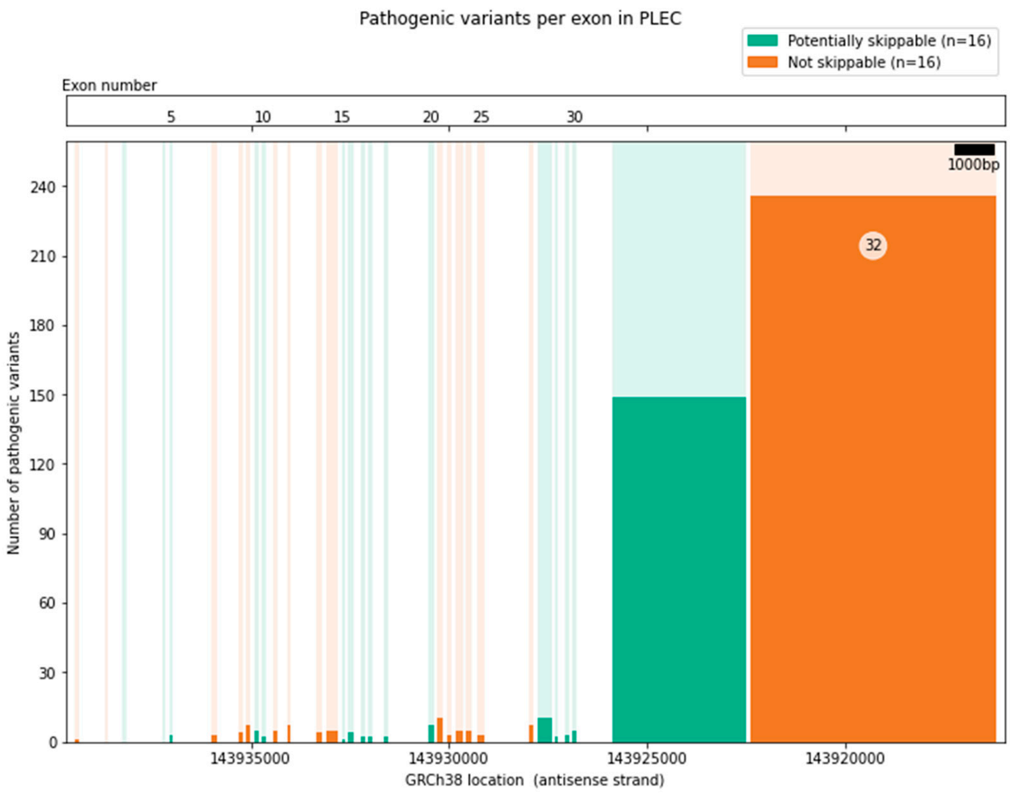

Supplemental Figure S9. *PLEC* Schematic representation of the genomic structure of *PLEC* and pathogenic variants per exon. Light-green bars indicate in-frame (potentially skippable) exons, light-orange bars out-of-frame (non-skippable) exons. Exons are depicted to scale, scale-bar represents 1000 bps. Projected on top of each exon as dark-green and dark-orange bars are the number of pathogenic variants per exon, the height of each bar indicates the number of variants per exon. In the top bar the exon numbers.

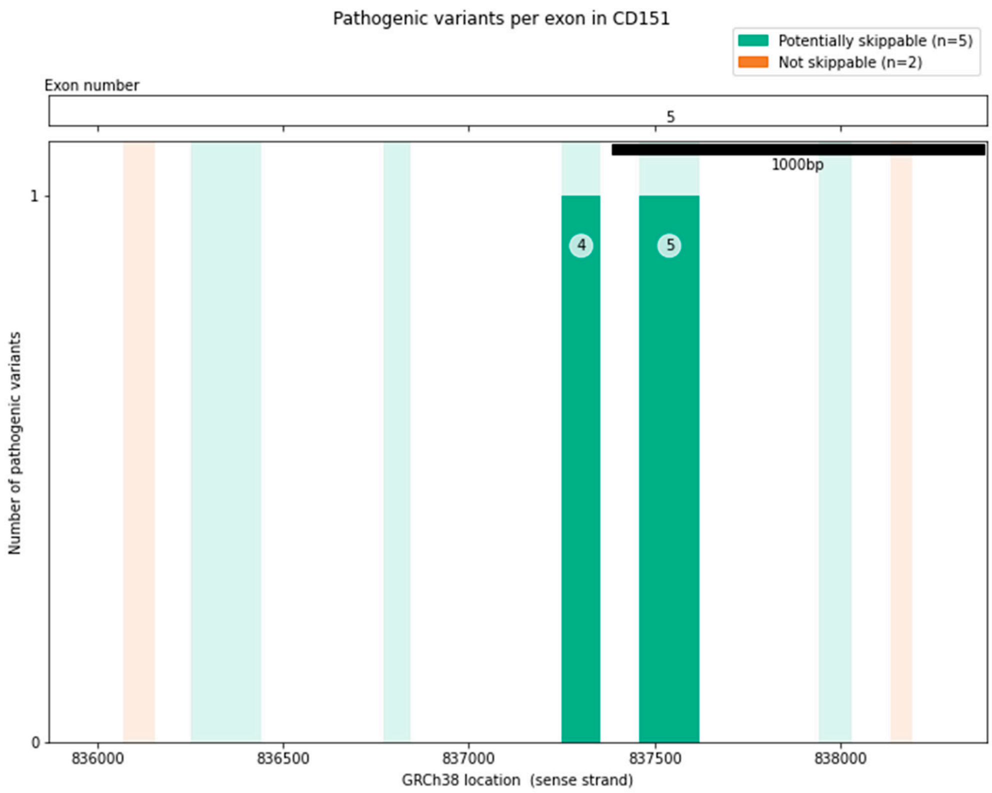

Supplemental Figure S10. Schematic representation of the genomic structure of *CD151* and pathogenic variants per exon. Light-green bars indicate in-frame (potentially skippable) exons, light-orange bars out-of-frame (non-skippable) exons. Exons are depicted to scale, scale-bar represents 1000 bps. Projected on top of each exon as dark-green and dark-orange bars are the number of pathogenic variants per exon, the height of each bar indicates the number of variants per exon. In the top bar the exon numbers.

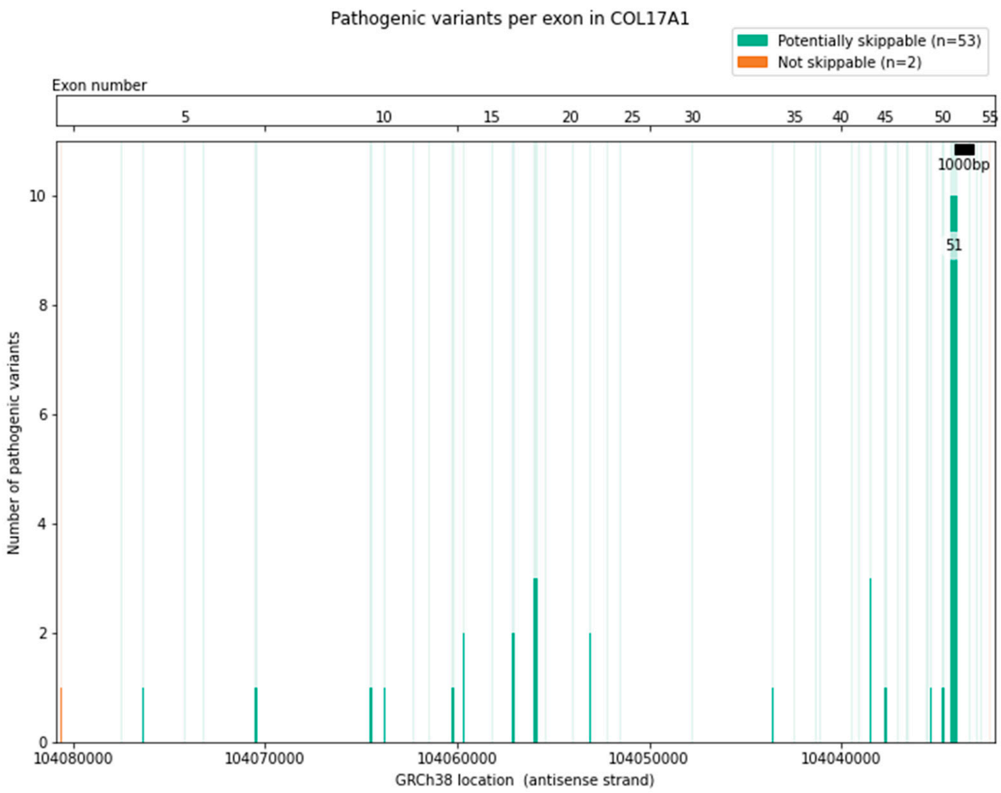

Supplemental Figure S11. Schematic representation of the genomic structure of *COL17A1* and pathogenic variants per exon. Light-green bars indicate in-frame (potentially skippable) exons, light-orange bars out-of-frame (non-skippable) exons. Exons are depicted to scale, scale-bar represents 1000 bps. Projected on top of each exon as dark-green and dark-orange bars are the number of pathogenic variants per exon, the height of each bar indicates the number of variants per exon. In the top bar the exon numbers.

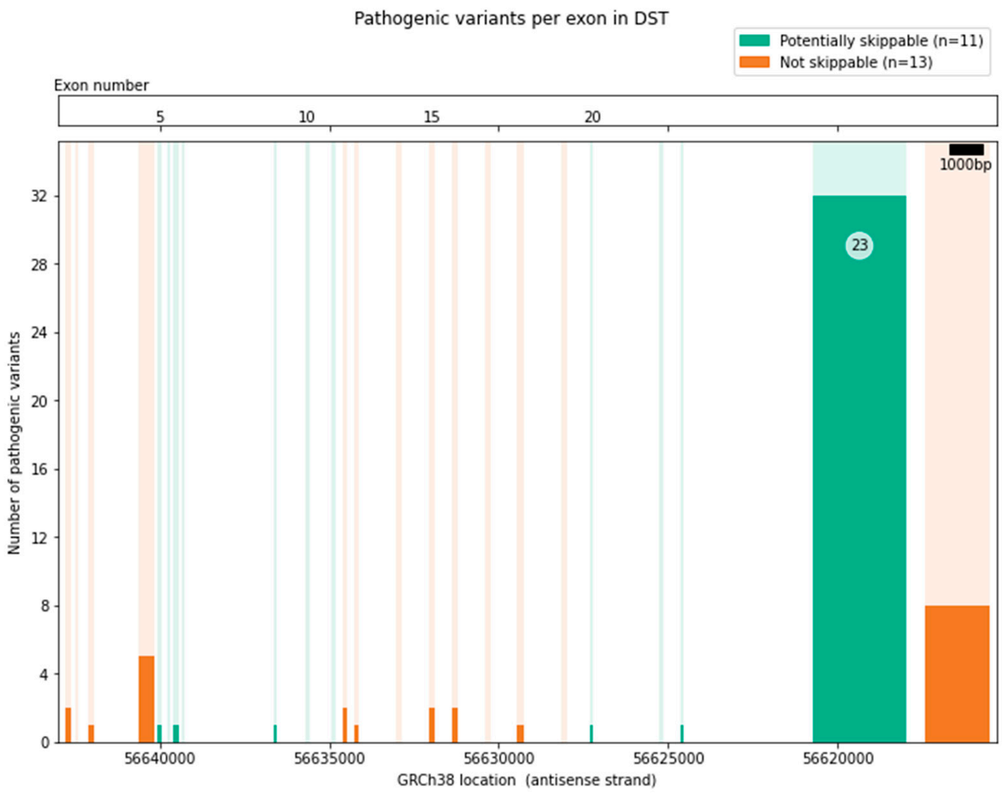

Supplemental Figure S12. Schematic representation of the genomic structure of *DST* and pathogenic variants per exon. Light-green bars indicate in-frame (potentially skippable) exons, light-orange bars out-of-frame (non-skippable) exons. Exons are depicted to scale, scale-bar represents 1000 bps. Projected on top of each exon as dark-green and dark-orange bars are the number of pathogenic variants per exon, the height of each bar indicates the number of variants per exon. In the top bar the exon numbers.

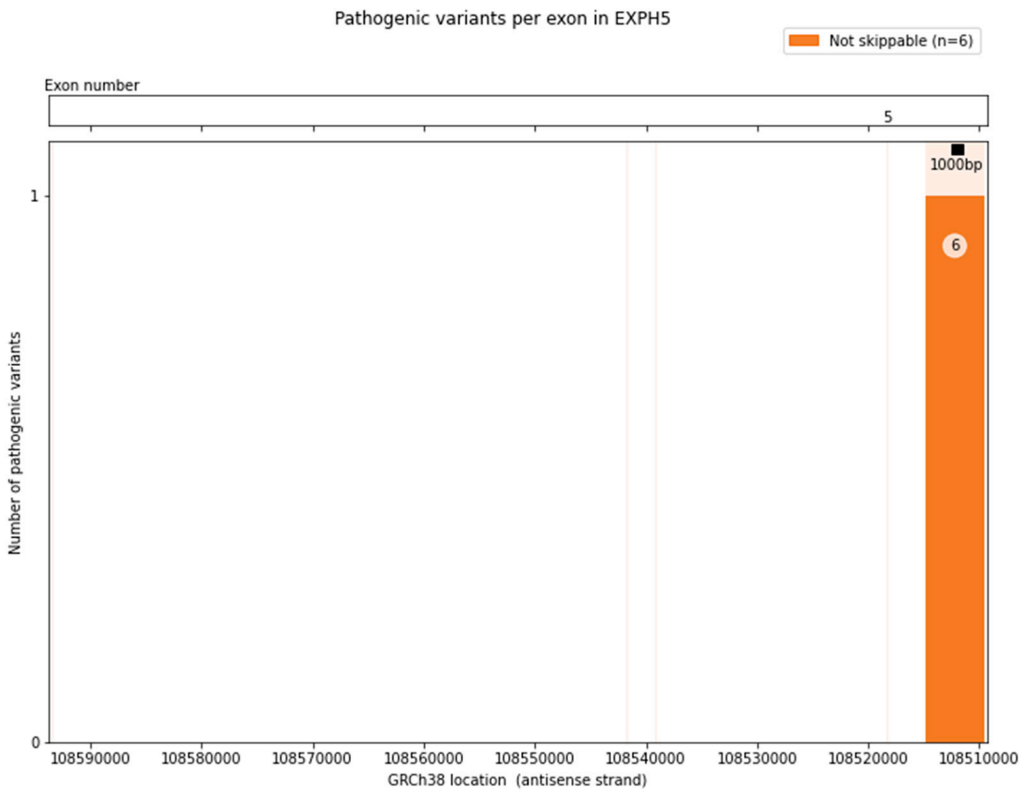

Supplemental Figure S13. Schematic representation of the genomic structure of *EXPH5* and pathogenic variants per exon. Light-green bars indicate in-frame (potentially skippable) exons, light-orange bars out-of-frame (non-skippable) exons. Exons are depicted to scale, scale-bar represents 1000 bps. Projected on top of each exon as dark-green and dark-orange bars are the number of pathogenic variants per exon, the height of each bar indicates the number of variants per exon. In the top bar the exon numbers.

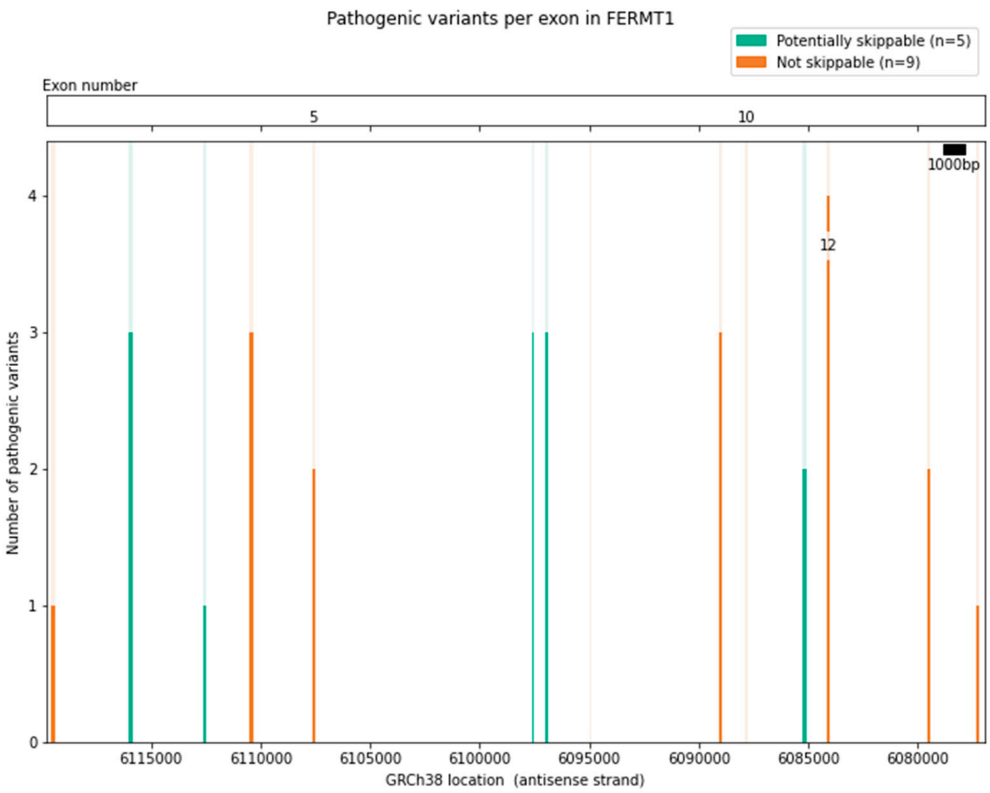

Supplemental Figure S14. Schematic representation of the genomic structure of *FERMT1* and pathogenic variants per exon. Light-green bars indicate in-frame (potentially skippable) exons, light-orange bars out-of-frame (non-skippable) exons. Exons are depicted to scale, scale-bar represents 1000 bps. Projected on top of each exon as dark-green and dark-orange bars are the number of pathogenic variants per exon, the height of each bar indicates the number of variants per exon. In the top bar the exon numbers.

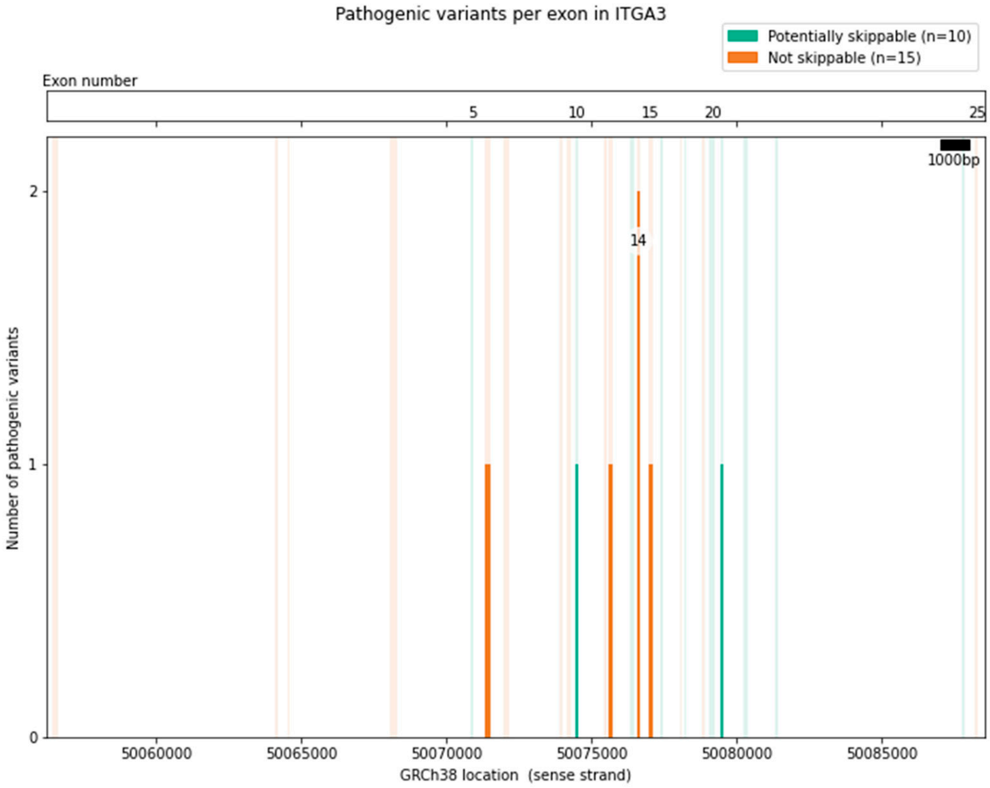

Supplemental Figure S15. Schematic representation of the genomic structure of *ITGA3* and pathogenic variants per exon. Light-green bars indicate in-frame (potentially skippable) exons, light-orange bars out-of-frame (non-skippable) exons. Exons are depicted to scale, scale-bar represents 1000 bps. Projected on top of each exon as dark-green and dark-orange bars are the number of pathogenic variants per exon, the height of each bar indicates the number of variants per exon. In the top bar the exon numbers.
